# Longitudinal neurobehavioral profiles in children and young adults with *PTEN* hamartoma tumor syndrome and reliable methods for assessing neurobehavioral change

**DOI:** 10.1186/s11689-022-09468-4

**Published:** 2023-01-14

**Authors:** Robyn M. Busch, Thomas W. Frazier II, Claire Sonneborn, Olivia Hogue, Patricia Klaas, Siddharth Srivastava, Antonio Y. Hardan, Julian A. Martinez-Agosto, Mustafa Sahin, Charis Eng

**Affiliations:** 1 Department of Neurology, Neurological Institute, Cleveland Clinic, Cleveland, OH USA; 2 Epilepsy Center, Neurological Institute, Cleveland Clinic, Cleveland, OH USA; 3 Genomic Medicine Institute, Lerner Research Institute, Cleveland Clinic, OH Cleveland, USA; 4 Department of Psychology, John Carroll University, University Heights, OH USA; 5 Center for Neurological Restoration, Neurological Institute, Cleveland Clinic, Cleveland, OH USA; 6 Department of Quantitative Health Sciences, Lerner Research Institute, Cleveland Clinic, Cleveland, OH USA; 7 Department of Neurology, Rosamund Stone Zander Translational Neuroscience Center, Boston Children’s Hospital, Harvard Medical School, Boston, MA USA; 8 Psychiatry and Behavioral Sciences, Stanford University Medical Center, Stanford, CA USA; 9 Department of Human Genetics, University of California Los Angeles, Los Angeles, CA USA; 10 Taussig Cancer Institute, Cleveland Clinic, Cleveland, OH USA; 11 Department of Genetics and Genome Sciences, Case Western Reserve University, Cleveland, OH USA

**Keywords:** PTEN, PTEN hamartoma tumor syndrome, Autism spectrum disorder, Cognition, Behavior, Reliable change indices, Standardized regression-based change scores

## Abstract

**Background:**

Individuals with *PTEN* hamartoma tumor syndrome (PHTS) demonstrate a distinct neurobehavioral profile suggesting primary disruption of frontal lobe symptoms, with more severe cognitive deficits in those with associated autism spectrum disorder (ASD) that extend to other areas of neurobehavioral function as well (e.g., adaptive behavior, sensory deficits). The current study sought to characterize longitudinal neurobehavioral profiles in individuals with PHTS who completed serial assessments (2–3 evaluations) over a 2-year time period.

**Methods:**

Comprehensive neurobehavioral evaluations were conducted on 92 participants (age range 6–21) with PHTS and/or ASD. Spaghetti plots and linear mixed effects models were used to visualize the individual patient profiles and group trends and examine the group differences in cognitive/behavioral test scores over time. Practice-adjusted reliable change indices (RCIs) and standardized regression-based change scores (SRBs) were calculated for those measures in the battery with adequate sample sizes and test–retest reliabilities for future use in assessing neurobehavioral change in children and young adults with PHTS.

**Results:**

Wide individual differences were observed at baseline across all measures. Encouragingly, baseline differences between patient groups persisted at the same magnitude over a 2-year time period with no differences in longitudinal neurobehavioral profiles within any one group. Test–retest reliabilities were generally high, ranging from 0.62 to 0.97, and group mean change from baseline to 12 months was small (range − 3.8 to 3.7). A Microsoft Excel calculator was created that clinicians and researchers can use to automatically calculate RCI and SRB thresholds at both 80% and 90% confidence intervals using test scores from a given child or young adult with PHTS.

**Conclusions:**

Our results suggest that the neurobehavioral phenotypes observed in individuals with PHTS remain relatively stable over time, even in those with ASD. The RCIs and SRBs provided can be used in future research to examine patient outcomes at the individual level as well as to detect negative deviations from the expected trajectory that can be used to inform intervention strategies.

**Supplementary Information:**

The online version contains supplementary material available at 10.1186/s11689-022-09468-4.

## Background

*PTEN* (OMIM + 601728) is a tumor suppressor gene located on 10q23.3 that plays a role in heritable and sporadic malignancies as well as in autism spectrum disorder (ASD) with macrocephaly. Irrespective of the clinical syndrome (e.g., Cowden, Bannayan-Riley-Ruvalcaba) and presence or absence of ASD, individuals carrying germline *PTEN* mutations are umbrellaed under the term *PTEN* hamartoma tumor syndrome (PHTS) [1, 2].

Over the past decade, research has demonstrated a rather consistent pattern of cognitive deficits suggesting frontal lobe systems dysfunction in both children and adults with PHTS, regardless of clinical phenotype [3, 4]. Specifically, deficits in attention, impulsivity, reaction time, executive function, processing speed, memory recall, and motor coordination have all been documented [3, 4]. Not surprisingly, however, the cognitive deficits observed in those with an ASD diagnosis (*PTEN*-ASD) are more severe than those observed in individuals with *PTEN* mutations without ASD (*PTEN*-No ASD) and extend to other areas of neurobehavioral function, including adaptive behavior and sensory deficits [5]. Interestingly, despite similarities in the core symptoms of ASD, individuals with *PTEN*-ASD have lower clinical ratings of autism severity and demonstrate more sensory abnormalities than those with macrocephalic ASD who do not harbor *PTEN* mutations (Macro-ASD) [5].

While there appears to be a rather consistent cognitive profile in PHTS and a distinct neurobehavioral phenotype in those with associated ASD, the developmental trajectory of cognitive and neurobehavioral functioning in these individuals is unclear as all research to date has been cross-sectional. To address this knowledge gap, the goal of the current study was to examine the longitudinal neurobehavioral trajectories in a large, prospective cohort of individuals with PHTS and/or ASD with macrocephaly and to develop reliable change indices (RCIs) and standardized regression-based change scores (SRBs) that can be used to assess clinically meaningful neurobehavioral changes in individuals with PHTS. Based on the existing literature on ASD, we hypothesized that the ASD groups would show score declines over time due to a failure to achieve the same developmental gains as their neurotypical peers.

## Methods

### Participants

Participants were recruited from one of four large tertiary medical centers in the USA (Cleveland Clinic, Boston Children’s Hospital, Stanford University Medical Center, and University of California, Los Angeles) as part of a multicenter prospective study to examine the natural history of autism and germline heterozygous *PTEN* mutations (clinicaltrials.gov: NCT02461446). Participants were included in this longitudinal sub-investigation if they met the following inclusion criteria: (1) age 3–21 years, (2) confirmed heterozygous mutation in *PTEN* and/or confirmed diagnosis of ASD (based on the consensus of Autism Diagnostic Observation Schedule-2 and expert clinician evaluation), (3) English as the primary language, and (4) completed baseline and at least one follow-up neurobehavioral assessment. Informed consent was obtained from adult participants and/or a parent or legal guardian. Participants aged 7 and older with the cognitive capability to do so provided assent for study participation.

Of 109 participants enrolled in the overarching study, seven were excluded because they did not have a history of ASD or a *PTEN* mutation, and 10 were excluded due to study withdrawal or loss to follow-up following baseline assessment. The remaining 92 participants were included and grouped into one of three categories based on clinical diagnosis and *PTEN* genotype: *PTEN*-ASD (*n* = 36; *PTEN* mutation with ASD), *PTEN*-no ASD (*n* = 26; *PTEN* mutation without ASD), or Macro-ASD (*n* = 30; ASD with macrocephaly but without *PTEN* mutation). Baseline assessments were completed between 8/2015 and 11/2019.

### *PTEN* genotyping

The Genomic Medicine Biorepository within the Genomic Medicine Institute at Cleveland Clinic extracted germline genomic DNA and performed PCR-based LightCycler mutation scanning and semi-automated PCR-based Sanger sequencing (ABI3730xl in Genomics Core Facility) of exons 1 through 9 and flanking intronic regions as per routine in the Eng lab since 1997 to reveal germline intragenic mutations in exons and splice sites. Novel variants identified were examined for the presence and frequency in 350 sex- and ancestry-matched population controls. To identify promotor variants, Sanger sequencing was utilized to conduct PCR-based sequence analysis of the extended promoter region. Identified promoter variants were then subjected to reporter assay as well as routine function interrogation. Large deletions and rearrangements were identified using multiplex ligation-dependent probe amplification.

### Measures

All participants completed at least two neuropsychological assessments that included both cognitive and behavioral measures. The cognitive battery included measures of global cognitive ability, working memory, processing speed, language, and visuospatial skills [5]. Given anticipated intellectual impairment in a subset of cases, executive functions and motor coordination were inferred from parent/guardian ratings on standardized questionnaires rather than measured directly. Caregivers also completed several questionnaires to assess autism symptoms, behavioral difficulties, sensory processing, and adaptive functioning. All measures were administered and scored in accordance with published test manuals using age- and/or sex-corrected norms as available/appropriate. Not all participants completed all study measures. Incomplete evaluations were largely due to the patient’s inability to tolerate testing or to time constraints. Additional file 1: Table S1 provides a list of the specific cognitive and behavioral measures employed. Higher cognitive scores reflect better cognitive performance for all measures except the Behavior Rating Inventory of Executive Function (BRIEF). Conversely, higher scores on the autism, sensory, and behavioral measures reflect greater symptom severity/behavioral difficulties, with the exception of the Short Sensory Profile (SSP).

### Statistical analyses

Baseline descriptive statistics were calculated for the three study groups and are summarized in Table 1. Differences in the proportion of missense versus truncating mutation types between *PTEN*-ASD and *PTEN*-no ASD were examined using chi-square analyses.

**Table 1:** Demographic characteristics of the study groups

	*PTEN*-ASD, *n* = 36	*PTEN*-no ASD, *n* = 26	Macro-ASD, *n* = 30	*P* value
Age^a^	9.0 (4.8) Range 3–19	8.9 (4.3) Range 3–18	11.5 (5.2) Range 4–21	.062
Sex				
Female Male	8 (22.2) 28 (77.8)	11 (42.3) 15 (57.7)	4 (13.3) 26 (86.7)	.039
Race				
White/Caucasian Black/African American Asian Multiracial Unknown/not reported	30 (83.3) 1 (2.8) – 3 (8.3) 2 (5.6)	21 (80.8) – 2 (7.7) 2 (7.7) 1 (3.8)	17 (56.7) 1 (3.3) 5 (16.7) 7 (23.3) –	.035
*PTEN* mutation status				
Missense Truncating	17 (54.8) 11 (35.5)	7 (28.0) 15 (60.0)	– –	.044 .067

*ASD* autism spectrum disorder

^a^Values presented as mean (standard deviation). All other values in table are presented as number (percentage)

#### Longitudinal neurobehavioral profiles

Spaghetti plots were used to visualize longitudinal data and include individual patient profiles as well as group trend lines. Linear mixed effects models (LMM) were used to determine whether the change in cognitive/behavioral scores over time differed among the groups. LMM are flexible extensions of linear regression that allow for unbalanced data; thus, all data could be retained, even if a subject missed a study visit. Each model included a random intercept for the subject and slope variance was evaluated but was not significant in all models and therefore was not retained. Group and time were fixed effects, and the coefficient of interest was the group-by-time interaction. Models applied a first-order autoregressive correlation structure, wherein observations closer to one another in time are assumed to be more related to one another than observations separated by a larger time window. Secondary analyses explored whether age, sex, race (white versus nonwhite), or baseline verbal, non-verbal, or full-scale IQ contributed to change over time. Each covariate of interest was considered both as a potential confounder and effect modifier for group change over time. Analyses were two-sided with an alpha of 0.05 and conducted using SAS Studio v.3.3. SAS code is available upon request.

#### Reliable change indices

For individuals with *PTEN* mutations (*n* = 62), practice-adjusted RCI cutoff scores were calculated for each of the neurobehavioral measures in the test battery according to the methods outlined by Jacobson and Truax [6]. Test–retest reliability coefficients were computed for each of the measures, and the standard error of measurement was used to calculate the standard error of the difference (SE_diff_), where SE_diff_ = √2(SEM)^2^. As noted by Jacobson and Truax, the SE_diff_ describes “the spread of the distribution of change scores that would be expected if no actual change had occurred” (p. 14), that is based solely on chance fluctuations in test scores across time [6]. Next, confidence intervals were established at 80% and 90% by multiplying the SE_diff_ by ± 1.28 and ± 1.64, respectively. This provided two different distribution ranges of change scores, with the 90% confidence interval offering a more conservative estimate of test–retest change and the 80% confidence interval a more liberal estimate. The resulting cutoff score ranges were then adjusted for practice effects as previously described in the literature [7, 8﻿]. The average practice effects were determined by calculating the mean change (i.e., time 2 mean minus time 1 mean) for each cognitive measure. These practice effects were then added to the confidence intervals in order to center the range of cutoff scores around the average test–retest practice. Score changes outside of these confidence intervals are considered uncommon in children and young adults with PHTS who have not experienced a new medical event or undergone any medical interventions during the test–retest interval.

#### Standardized regression-based change scores

Linear regression analyses were conducted to predict 12-month retest scores for certain neuropsychological measures for the subset of participants with *PTEN* mutations (*n* = 62). The baseline test score was considered as the main predictor, and age, diagnosis (ASD vs. no ASD), and intellectual disability status (intellectual disability vs. no intellectual disability) were considered as other modifying predictors. Best subsets selection was used for variable selection [9]. All potential predictors were entered into an exhaustive subset selection for models with one, two, and three predictors. The best model with each of one, two, and three predictors was selected. Then, the model with the highest *R*^2^ value was chosen from the three models. This was compared to the model using only the baseline test score predictor. If the improvement in *R*^2^ from the baseline-only to the best subset-selected model was less than 0.05, then the baseline-only model was chosen for simplicity of interpretation. The residual versus fitted value plots were visually checked for each model to confirm linearity and homoscedasticity.

RCIs and SRB change scores were conducted using R version 3.6.3. Because RCI and SRB creation is not hypothesis-testing, *p*-values are not created nor presented. R code is available upon request.

## Results

### Demographic characteristics

There were no significant age differences between the three study groups. Consistent with the known sex ratio in ASD, there was a smaller proportion of males in the *PTEN*-No ASD group compared to the two ASD groups, and the Macro-ASD group was more racially diverse than the PHTS groups [Table 1].

### Genetic analyses

A summary of the germline *PTEN* variants observed in the study cohort is provided in Additional file 2: Table S2. There were modest differences between the groups in terms of the type of variants observed. Specifically, there was a larger proportion of patients with missense mutations (54.8% vs. 28.0%, *p* = 0.044) and fewer patients with truncating variants (35.5% vs. 60.0%, *p* = 0.067) in the *PTEN*-ASD group compared to *PTEN*-no ASD group.

### Longitudinal neurobehavioral profiles

**Fig. 1:**
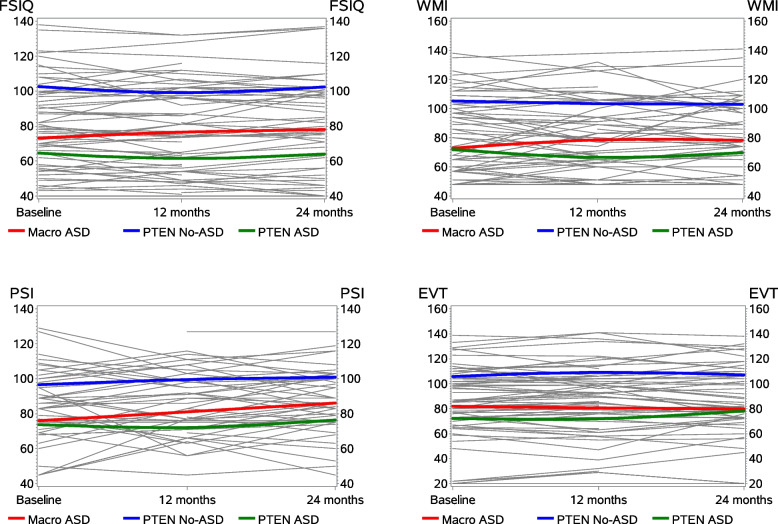
Longitudinal performance on cognitive measures. Spaghetti plots depicting cognitive performance over time on select cognitive measures. **A** Stanford-Binet Intelligence Scales Full Scale IQ. **B** Sanford-Binet Intelligence Scales Working Memory Index. **C** Wechsler Intelligence Scale for Children – Fifth Edition Processing Speed Index. **D** Expressive Vocabulary Test. Each gray line represents a single subject, while each colored line represents the group trend over time (blue: *PTEN*-no ASD; red: Macro-ASD; green: *PTEN*-ASD). Fragmented subject lines indicate the partial follow-up data

The results for some of the key cognitive measures are depicted in Fig. 1a–d. Similar patterns were observed on all other measures examined. Wide individual differences were observed at baseline across all measures. However, all groups remained stable over time on all cognitive and behavioral measures (all group slopes near zero). There were no interactions between group and time (all *p* > 0.05, linear mixed effects models), and there was minimal variance in individual trajectories.

### Reliable change indices for PHTS

A summary of baseline and retest mean scores and standard deviations along with the mean change scores and test–retest reliabilities for each test is presented in Table 2. The mean change scores were slightly variable across the neuropsychological measures, with a range of − 3.8 to 3.7. There were no categories of tests that seemed to have especially high mean change scores. The test–retest reliabilities were relatively high for measures in the battery, with coefficients ranging from 0.40 to 0.97. The Tactile Sensitivity subscale of the Short Sensory Profile was the only measure with a test–retest reliability score under 0.5 (i.e., 0.40). Given this low test–retest reliability, RCIs and SRBs were not calculated for this measure. All other measures had coefficients in the range of 0.62 to 0.97. RCIs and SRBs were also not calculated for the Motor Skills subscale of the Vineland Adaptive Behavior Scale-Second Edition, given the small sample size for this measure (*n* = 16).

**Table 2:** Test–retest means, standard deviations, mean difference scores, and test–retest reliabilities

	Number	Time 1 mean (SD)	Time 2 mean (SD)	Mean change	Test–retest reliability
Stanford-Binet Intelligence Scales – Fifth Edition					
Full-scale IQ	35	82.0 (27.1)	80.5 (27.0)	− 1.6	.93
Verbal IQ	35	83.3 (27.8)	81.4 (27.3)	− 1.9	.93
Nonverbal IQ	37	81.1 (26.6)	80.0 (26.3)	− 1.1	.93
Working memory	35	88.1 (25.6)	85.3 (26.2)	− 2.8	.93
Wechsler Processing Speed Index*	28	85.6 (23.3)	86.1 (18.9)	0.4	.74
Peabody Picture Vocabulary Test	35	86.6 (23.2)	85.5 (34.5)	− 1.1	.90
Expressive Vocabulary Test	37	86.7 (30.1)	88.0 (30.0)	1.3	.97
Behavior Rating Inventory of Executive Function					
Global Executive Composite	28	61.0 (14.1)	59.0 (14.1)	− 2.0	.86
Behavioral Regulation Index	24	55.8 (13.8)	56.5 (16.6)	0.7	.83
Inhibit	29	54.3 (11.8)	56.8 (15.2)	2.5	.62
Shift	29	58.5 (15.1)	56.7 (15.2)	− 1.8	.89
Emotional control	29	54.8 (13.8)	53.4 (13.4)	− 1.4	.83
Metacognition index	23	61.5 (13.9)	59.0 (13.5)	− 2.4	.63
Initiate	24	61.5 (14.2)	57.7 (13.2)	− 3.8	.81
Working memory	29	63.0 (16.0)	62.3 (14.3)	− 0.7	.76
Plan/organize	28	61.3 (15.7)	58.0 (14.8)	− 3.3	.64
Organization of materials	24	52.0 (12.3)	52.0 (11.9)	0.0	.78
Monitor	22	59.2 (13.6)	59.2 (15.0)	0.0	
Visual-Motor Integration	36	78..0 (20.4)	78.7 (21.3)	0.7	.90
Developmental Coordination Disorder Questionnaire	36	33.6 (15.6)	36.3 (14.6)	2.7	.88
Social Responsiveness Scale – Second Edition	35	66.6 (15.4)	66.6 (15.8)	0.0	.86
Repetitive Behavior Scale – Revised	36	21.1 (21.2)	17.9 (16.4)	− 3.2	.74
Child Behavior Checklist					
Externalizing	41	58.3 (12.9)	57 (12.5)	− 1.3	.82
Internalizing	41	47.8 (10.8)	49.7 (11.4)	− 1.9	.83
Total problems	41	56.7 (12.6)	56.9 (12.6)	0.2	.89
Short Sensory Profile					
Taste/smell sensitivity	34	14.8 (6.1)	14.6 (5.8)	− 0.2	.63
Movement sensitivity	34	11.6 (3.3)	11.7 (3.6)	0.1	.71
Under-responsive/seeks sensation	34	24.4 (8.7)	25.3 (8.2)	1.0	.81
Auditory filtering	34	19.8 (6.2)	20.1 (5.5)	0.3	.70
Low energy/weak	34	18.6 (8.9)	17.7 (8.7)	− 0.9	.83
Visual/auditory sensitivity	34	18.0 (5.5)	18.4 (4.7)	0.3	.74
Total	33	137.5 (32.2)	139.7 (28.7)	2.2	.75
Vineland Adaptive Behavior Scale – Second Edition					
Communication	34	82.1 (25.7)	83.0 (25.2)	0.9	.92
Daily living skills	34	80.6 (24.4)	81.3 (21.6)	0.7	.85
Socialization	32	83.7 (27.3)	84.9 (22.1)	1.3	.83
Adaptive behavior composite	29	78.4 (23.1)	78.7 (19.9)	0.3	.88
Externalizing	31	15.6 (2.7)	16.4 (3.3)	0.8	.63
Internalizing	31	19.1 (3.2)	19.3 (2.8)	0.3	.68
Receptive language	34	11.6 (4.5)	11.9 (4.6)	0.3	.75
Expressive language	34	11.4 (4.8)	11.8 (5.0)	0.4	.84
Written expression	34	12.8 (4.3)	12.6 (4.1)	− 0.1	.84
Personal	34	11.1 (4.6)	11.3 (4.2)	0.2	.69
Domestic	34	12.4 (4.0)	12.2 (3.7)	− .2	.82
Interpersonal Relationships	35	49.0 (18.8)	51.5 (16.6)	2.5	.89
Play and Leisure Time	33	36.0 (17.6)	39.7 (17.2)	3.7	.83
Coping Skills	33	33.6 (17.8)	34.1 (18.5)	0.5	.62

^*^Processing Speed Index standard scores from all available Wecshler scales were combined

Adjusted reliable change cut scores at both 80% and 90% confidence intervals are provided in Table 3 along with the correction value used to adjust for practice effects. The adjusted reliable change scores reported in Table 3 represent the cutoff values at or beyond which an observed change score would represent a clinically meaningful change, after adjusting for test–retest reliability and practice effects. For example, a child or young adult whose Working Memory Index improved by 12 standard score points from baseline to 1-year retest would show a clinically meaningful change falling outside of the 80% confidence interval, but not outside of the 90% confidence interval. A 6-point improvement, however, would be considered unremarkable in this patient, but rather would be thought to reflect the unreliability of the measure and typical practice effects.

**Table 3:** Adjusted reliable change indices (80% and 90% confidence intervals)

	Practice	Adjusted RC 80%	Adjusted RC 90%
Stanford-Binet Intelligence Scales – Fifth Edition^a^			
Full-scale IQ	− 2	− 14, 11	− 18, 15
Verbal IQ	− 2	− 15, 11	− 18, 15
Nonverbal IQ	− 1	− 14, 11	− 17, 15
Working memory	− 3	− 15, 10	− 19, 13
Wecshler Processing Speed Index^a*^	0	− 21, 22	− 27, 28
Peabody Picture Vocabulary Test^a^	− 1	− 20, 18	− 26, 24
Expressive Vocabulary Test^a^	1	− 8, 10	− 10, 13
Behavior Rating Inventory of Executive Function^b^			
Global Executive Composite	− 2	− 14, 10	− 12, 8
Behavioral Regulation Index	1	− 10, 11	− 12, 14
Inhibit	2	− 11, 16	− 14,19
Shift	− 2	− 11, 7	− 14, 10
Emotional control	− 1	− 16, 13	− 19, 17
Metacognition index	− 2	− 13, 8	− 16, 11
Initiate	− 4	− 19, 12	− 24, 16
Working memory	− 1	− 13, 12	− 17, 16
Plan/organize	− 3	− 18, 11	− 22, 15
Organization of materials	0	− 13, 13	− 17, 17
Monitor	0	− 11, 12	− 15, 15
Visual-Motor Integration^a^	1	− 11, 13	− 14, 16
Developmental Coordination Disorder Questionnaire^c^	3	− 7, 12	− 10, 15
Social Responsiveness Scale – Second Edition^b^	0	− 10, 10	− 13, 13
Repetitive Behavior Scale – Revised^c^	− 3	− 23, 16	− 28, 22
CBCL			
Externalizing	− 2	− 11, 8	− 14, 11
Internalizing	2	− 6, 10	− 9, 12
Total score	0	− 7, 8	− 10, 10
Short Sensory Profile^c^			
Taste/smell sensitivity	0	− 7, 6	− 9, 8
Movement sensitivity	0	− 3, 3	− 4, 4
Under-responsive/seeks sensation	1	− 6, 8	− 8, 10
Auditory filtering	0	− 6, 6	− 8, 8
Low energy/weak	− 1	− 7, 6	− 9, 8
Visual/auditory sensitivity	0	− 5, 5	− 6, 7
Total	2	− 27, 31	− 35, 39
Vineland Adaptive Behavior Scale – Second Edition^d^			
Communication	1	− 13, 14	− 16, 18
Daily living skills	1	− 17, 18	− 21, 23
Socialization	1	− 19, 22	− 25, 28
Adaptive behavior composite	0	− 14, 15	− 18, 19
Externalizing	1	− 2, 4	− 3, 5
Internalizing	0	− 3, 3	− 4, 4
Receptive	0	− 4, 4	− 5, 6
Expressive	0	− 3, 4	− 4, 5
Written	0	− 3, 3	− 4, 4
Personal	0	− 4, 5	− 6, 6
Domestic	0	− 3, 3	− 4, 4
Interpersonal Relationships	2	− 9, 14	− 12, 17
Play and Leisure Time	4	− 9, 17	− 13, 20
Coping Skills	1	− 13, 14	− 17, 18

Results are only reported for cognitive measures with test–retest reliabilities above .50. When interpreting an individual patient’s pattern of scores, test–retest differences falling at or below the lower limit of the RC interval or at or above the upper limit of the RC interval would be indicative of clinically meaningful change

*RC* reliable change

^a^Standard scores

^b^T-scores

^c^Raw scores

^d^v-scale scores

^*^Processing Speed Index standard scores from all available Wecshler scales were combined

### Standardized regression-based change scores for PHTS

For each regression model, coefficients for the intercept and predictors as well as adjusted *R*^2^ values, residual standard error, and degrees of freedom are reported in Table 4.

**Table 4:** Standard regression-based change score norms for the chosen models

Measure	Adj. *R*^2^	SE_est_	*C*	*β*_baseline_	*β*_age_	*β*_ID_	df
Stanford Binet Intelligence Scales – 5^th^ Edition^a^							
Full-scale IQ	0.8644	9.938	4.255	0.929	–	–	33
Verbal IQ	0.8649	9.665	5.287	0.921	–	–	35
Nonverbal IQ	0.8683	9.922	4.941	0.918	–	–	33
Working memory	0.8540	10.03	1.522	0.951	–	–	33
Peabody Picture Vocabulary Test^a^	0.9163	10.18	3.351	0.899	0.574	− 13.371	27
Expressive Vocabulary Test^a^	0.9431	7.152	4.144	0.967	–	–	35
Visual-Motor Integration^a^	0.8001	9.541	5.638	0.937	–	–	34
Social Responsiveness Scale Second Edition^b^	0.7323	8.176	7.854	0.882	–	–	33
CBCL							
Externalizing	0.6701	7.160	10.268	0.798	–	–	39
Internalizing	0.6793	6.456	7.993	0.872	–	–	39
Total score	0.7830	5.872	6.303	0.891	–	–	39
Vineland Adaptive Behavior Scale – Second Edition^a^							
Communication	0.8353	10.23	9.315	0.898	–	–	32
Daily living skills	0.709	11.66	20.916	0.749	–	–	32
Adaptive behavior composite	0.7672	9.618	19.141	0.759	–	–	27
Socialization	0.7803	11.84	44.082	0.609	-0.858	-14.359	38

R﻿esults are only reported for cognitive measures with models that were well-fit, based on visual assessment of residuals, and with test–retest reliabilities above .50. Numbers in parentheses after equations with modifiers represent the percent of variance accounted for by the cognitive measure alone

*Adj. R*^*2*^ amount of variance explained by the model adjusted for number of coefficients, *SE*_*est*_ standard error of the estimate, *C* constant; *B*_*baseline*_ unstandardized beta (slope) for baseline test score, *B*_*age*_ slope for age at baseline testing, *B*_*ID*_ slope for intellectual disability

^a^Standard scores

^b^T-scores

The coefficients in the table, along with the standard error of the estimate, can be used to calculate the standardized regression-based change score norms that predict the retest score. First, an estimate of the retest score is calculated using the coefficients for the regression. For example, for a child with a full-scale IQ score of 80, the regression estimate for the retest score is 4.255 + 80 × 0.929 which is 78.575. The difference between this predicted estimate and the actual score is then calculated and transformed into a *z*-score by dividing it by the standard error of the estimate. Say the child from this example had an actual retest score of 73 a year after baseline, then the *z*-score for this child would be (73 − 78.575)/9.938 which is − 0.56. *z*-scores with absolute values ≥ 1.28 are outside of the 80% confidence interval while *z*-scores with absolute values ≥ 1.64 fall outside of the 90% confidence interval. Scores outside the 80% and 90% confidence intervals indicate that the change in score is not likely due to chance or retest practice. The child in this example falls within both confidence intervals, and their retest score would not be considered unusual.

A Microsoft Excel calculator has been created to calculate RCI and SRB thresholds at both 80% and 90% confidence intervals using test scores from a given child or young adult with PHTS and is available from the corresponding author upon reasonable request.

## Discussion

This longitudinal neurobehavioral study demonstrates that baseline group differences in cognitive and behavioral function observed in our prior research [5] persist at the same magnitude over a 2-year time period with no group differences in longitudinal neurobehavioral profiles. Specifically, both *PTEN* groups (*PTEN*-ASD and *PTEN*-no ASD) show cognitive patterns suggesting primary involvement of frontal lobe systems, but those with ASD demonstrate more severe deficits in frontal lobe functions along with language, adaptive behavior, and sensory deficits not observed in the *PTEN*-No ASD group. While both ASD groups (*PTEN*-ASD and Macro-ASD) showed reduced performance across a broad range of cognitive measures, those with *PTEN*-ASD showed slower reaction times and more sensory abnormalities than those with Macro-ASD [5]. Our longitudinal data show that these patterns of group differences remain remarkably stable over serial assessments (i.e., 2–3 evaluations over a 2-year time frame) on all cognitive and behavioral measures examined. It should be noted that, while the present data suggest that the neurocognitive functions remain fairly stable in patients with PHTS (with and without ASD), this differs from studies of IQ in an idiopathic ASD population where IQ tended to increase from early childhood to adulthood and remained more variable than expected [10]. It is possible that this difference is simply due to the short observation period of the present study. Clearly, additional longitudinal follow-up of this cohort will be useful for better understanding the stability of IQ and other neurocognitive functions in the PHTS population.

Oftentimes, children with ASD will fail to show the same developmental gains as their same-aged neurotypical peers, resulting in either a drop in normative adaptive scores over time or barely keeping pace even in the context of intensive behavioral intervention [11–14]. Thus, it is somewhat encouraging that there do not appear to be any longitudinal changes in cognitive performance in any of the three study groups over time. In fact, this suggests that, at the group level, even the reductions in neurobehavioral skills seen in *PTEN*-ASD cases do not further decline with age. This suggests that common intervention strategies provided to *PTEN*-ASD cases, such as behavioral intervention, may be useful for sustaining growth, similar to what is seen in more impaired sub-groups of idiopathic ASD [12]. Although group trajectories remain stable, even for *PTEN*-ASD, analyses demonstrated substantial variability in the level of neurobehavioral function. Thus, it remains crucial that individuals with PHTS, particularly those with ASD, who show substantial reductions in cognitive and functional skills and/or behavior problems receive behavioral and educational interventions needed to help them maximize functioning. Furthermore, RCIs and SRBs can be used to identify cases showing deviations in expected development and guide additional intervention strategies to reduce the likelihood of further deviation from normative trajectories.

In addition to assessing longitudinal neurobehavioral changes at the group level, this rich dataset allowed us to develop RCIs and SRBs that clinicians can use to identify meaningful cognitive and behavioral changes at the individual level in children and adolescents with PHTS. The present data showed significant variability across patients in overall neurobehavioral levels but only minimal individual differences in change over time. However, it is important to note that minimal slope variability may be, in part, a function of the small sample size as a few individuals showed meaningful changes across time points. Regardless, there is always the potential that an individual patient will deviate from expected trajectories. RCI and SRB tools provide a mechanism for detecting these deviations and also a common metric that researchers can use in future studies to more accurately characterize cognitive outcomes in children and adolescents with PHTS. Importantly, the methods for assessing cognitive change provided here allow differentiation between changes in neurobehavioral function over time due to PHTS and changes that may result from associated medical events, interventions, and/or treatments, which cannot be accomplished using traditional methods (e.g., change scores).

Here, we provide two different metrics for assessing cognitive change (i.e., RCIs and SRBs) so that clinicians and researchers can select the method that best addresses their needs. RCI methodology determines the degree of individual change associated with test imprecision and practice effects and identifies the amount of test–retest change required in order to conclude that clinical change has occurred independent of measurement error. RCIs provide cutoff scores to identify meaningful change, requiring no additional calculation beyond test–retest difference scores. As such, they are quick and easy to apply to a patient’s or participant’s test results. However, it should be noted that RCIs do not correct for regression to the mean or other potential modifying factors.

SRB methodology, which is a bit more complicated to use, corrects for multiple confounding factors that RCIs do not. Statistically, SRBs correct for practice by using an individual’s baseline score as a predictor of retest performance. This provides a more accurate adjustment of practice effects than RCIs because practice can be estimated differently at different levels of baseline performance. SRBs also allow for the correction of other potential modifiers that may impact cognitive performance over time. Finally, SRBs convert changes in test scores to a common metric (i.e., *z*-scores) permitting direct comparison of cognitive change across a wide range of neuropsychological measures.

Studies comparing RCI to SRB methodologies suggest that predictive accuracy is similar for both measures [15, 16]. As a result, many clinicians and researchers prefer to utilize the easily employed RCI cutoffs rather than calculating SRBs for individual patients or participants. Regardless of the method preferred, we have created a Microsoft Excel calculator that calculates both RCIs and SRBs to facilitate the interpretation of cognitive change in individual patients across a wide range of cognitive and behavioral methods. This calculator is available from the corresponding author upon request.

It is interesting to note that some of the neurobehavioral measures in this study were associated with negative practice effects in our sample. Rather than showing the typical practice effects demonstrated by healthy children, children in our PHTS sample achieved lower test scores, on average, during repeat testing on some neurobehavioral measures. Similar findings, with a lack of typical practice effects, have been reported in other disorders that affect the central nervous system, like epilepsy [17, 18]. In our case, this may indicate that, despite relatively stable cognitive profiles at the group level, some children with PHTS may not be developing along the expected trajectory. Most of the measures in our battery are age-normed; therefore, if children with PHTS are not gaining skills at a rate comparable to healthy standardization samples, their scores on these measures will decline over time. Alternatively or additionally, the measures with negative practice effects are all sensitive to frontal lobe functioning, which is known to be affected in PHTS, and may indicate that children with PHTS do not show typical gains in frontal lobe functions compared to their same-aged neurotypical peers. Regardless of the reason for the observed negative practice effects, we accounted for this in our RCI and SRB development by centering intervals around typical changes in scores, regardless of whether the practice effects were positive or negative.

## Limitations

There are several study limitations that should be mentioned. Although the sample sizes are relatively large considering PHTS is a rare disease, the group sizes are small statistically speaking and future research with larger samples will be required to fully understand the spectrum and longitudinal trajectories of neurobehavioral functioning in these individuals. Our data are also limited to a 2-year time frame; longer follow-up periods will be needed to truly understand the potential developmental impact of PHTS over the lifespan and how clinical interventions might alter the neurobehavioral course over time. Given the small sample size, it was also impossible to control for treatment interventions that the participants may have been receiving (e.g., behavioral or occupational therapies). Sample size also limited our ability to examine whether the measures collected are truly measuring the same neurobehavioral constructs in PHTS. Future research should examine the measurement invariance in genetic syndromes, including PHTS, to ensure that these measures are evaluating the same constructs as in the (largely) neurotypical populations where they were developed. Future research with larger samples will also be needed in order to tease apart the impact of interventions and experience on neurobehavioral trajectories versus the natural development of these functions over time and to subdivide *PTEN*-ASD into those with and without intellectual disability to better understand the differential impact of ASD versus intellectual disability on longitudinal trajectories.

## Conclusions

The longitudinal data described in this study suggest that patterns of neurobehavioral function observed in children and young adults with PHTS and/or ASD remain stable over a 2-year time frame with no group differences in longitudinal neurobehavioral profiles. RCIs and SRBs were developed, and an Excel calculator was provided that clinicians and/or researchers can use to identify reliable individual patient change over time to detect negative deviations from the expected trajectory and to inform intervention strategies to maximize neurobehavioral function.

## Supplementary Information

**Additional file 1.**

**Additional file 2.**

**Additional file 3.**

## Data Availability

The datasets analyzed in the current study are not publicly available because of restricted access, but further information about the datasets is available from the corresponding author upon reasonable request.

## Abbreviations

ASD: Autism spectrum disorder
PHTS: PTEN hamartoma tumor syndrome
RCIs: Reliable change indices
SRBs: Standardized regression-based change scores
