# Supplementary figures and images for: Longitudinal neurobehavioral profiles in children and young adults with PTEN hamartoma tumor syndrome and reliable methods for assessing neurobehavioral change
Source: J Neurodev Disord. 2023 Jan 14;15:3. doi: 10.1186/s11689-022-09468-4 (PMC9840250; doi:10.1186/s11689-022-09468-4)

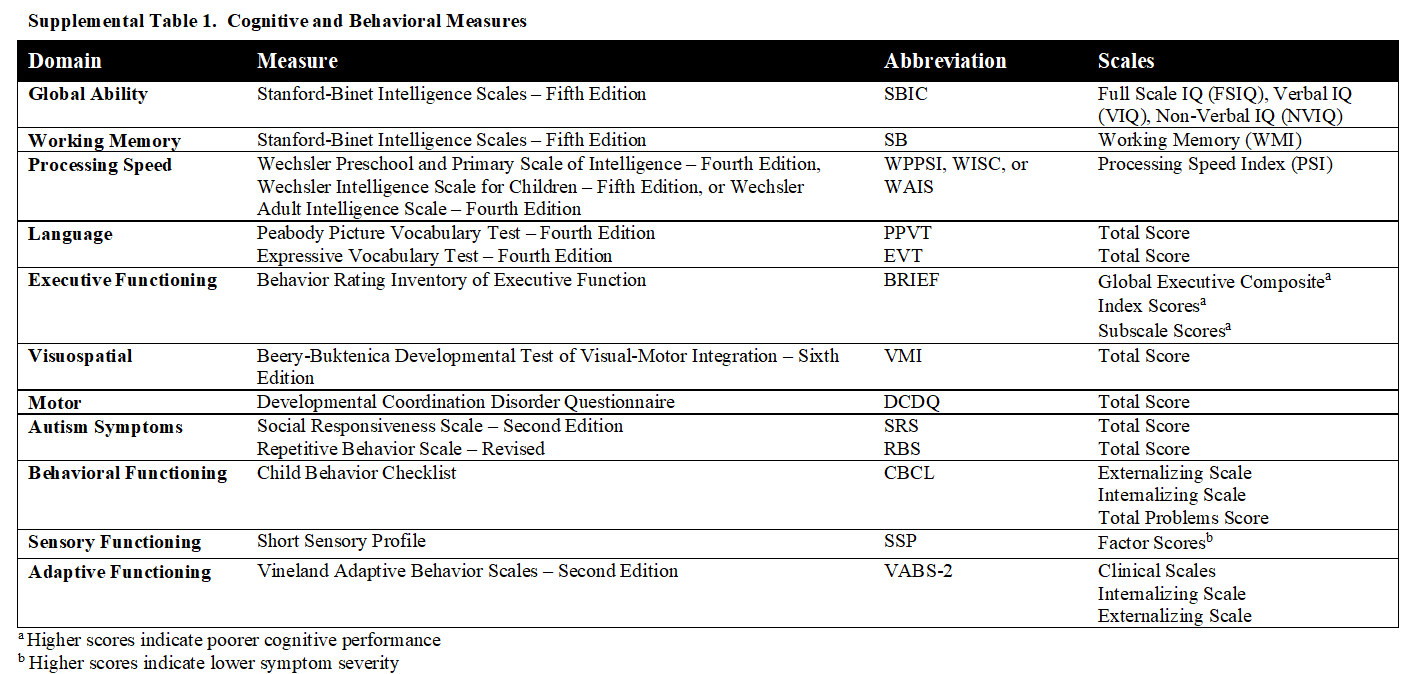

Supplement: Supplementary file 1 — Additional file 1. [file 11689_2022_9468_MOESM1_ESM.docx]
